# Linking Nitrogen Load to the Structure and Function of Wetland Soil and Rhizosphere Microbial Communities

**DOI:** 10.1128/mSystems.00214-17

**Published:** 2018-01-30

**Authors:** Eric R. Hester, Sarah F. Harpenslager, Josepha M. H. van Diggelen, Leon L. Lamers, Mike S. M. Jetten, Claudia Lüke, Sebastian Lücker, Cornelia U. Welte

**Affiliations:** a Department of Microbiology, Radboud University, Nijmegen, The Netherlands; b Department of Aquatic Ecology and Environmental Biology, Radboud University, Nijmegen, The Netherlands; c School of Biological and Chemical Sciences, Queen Mary University, London, United Kingdom; d B-WARE Research Centre, Nijmegen, The Netherlands; Argonne National Laboratory

**Keywords:** *Acidobacteria*, *Juncus acutiflorus*, *Opitutales*, *Sphingobacteriales*, greenhouse gas, metagenomics, microbial community function, nitrogen, nitrogen metabolism, wetlands

## Abstract

Wetland ecosystems are important reservoirs of biodiversity and significantly contribute to emissions of the greenhouse gases CO_2_, N_2_O, and CH_4_. High anthropogenic nitrogen (N) inputs from agriculture and fossil fuel combustion have been recognized as a severe threat to biodiversity and ecosystem functioning, such as control of greenhouse gas emissions. Therefore, it is important to understand how increased N input into pristine wetlands affects the composition and activity of microorganisms, especially in interaction with dominant wetland plants. In a series of incubations analyzed over 90 days, we disentangled the effects of N fertilization on the microbial community in bulk soil and the rhizosphere of *Juncus acutiflorus*, a common and abundant graminoid wetland plant. We observed an increase in greenhouse gas emissions when N is increased in incubations with *J. acutiflorus*, changing the system from a greenhouse gas sink to a source. Using 16S rRNA gene amplicon sequencing, we determined that the bacterial orders *Opitutales*, subgroup 6 *Acidobacteria*, and *Sphingobacteriales* significantly responded to high N availability. Based on metagenomic data, we hypothesize that these groups are contributing to the increased greenhouse gas emissions. These results indicated that increased N input leads to shifts in microbial activity within the rhizosphere, altering N cycling dynamics. Our study provides a framework for connecting environmental conditions of wetland bulk and rhizosphere soil to the structure and metabolic output of microbial communities.

**IMPORTANCE** Microorganisms living within the rhizospheres of wetland plants significantly contribute to greenhouse gas emissions. Understanding how microbes produce these gases under conditions that have been imposed by human activities (i.e., nitrogen pollution) is important to the development of future management strategies. Our results illustrate that within the rhizosphere of the wetland plant *Juncus acutiflorus*, physiological differences associated with nitrogen availability can influence microbial activity linked to greenhouse gas production. By pairing taxonomic information and environmental conditions like nitrogen availability with functional outputs of a system such as greenhouse gas fluxes, we present a framework to link certain taxa to both nitrogen load and greenhouse gas production. We view this type of combined information as essential in moving forward in our understanding of complex systems such as rhizosphere microbial communities.

## Abstract (precis)

Microorganisms living within the rhizospheres of wetland plants significantly contribute to greenhouse gas emissions. Understanding how microbes produce these gases under conditions that have been imposed by human activities (i.e., nitrogen pollution) is important to the development of future management strategies. Our results illustrate that within the rhizosphere of the wetland plant *Juncus acutiflorus*, physiological differences associated with nitrogen availability can influence microbial activity linked to greenhouse gas production. By pairing taxonomic information and environmental conditions like nitrogen availability with functional outputs of a system such as greenhouse gas fluxes, we present a framework to link certain taxa to both nitrogen load and greenhouse gas production. We view this type of combined information as essential in moving forward in our understanding of complex systems such as rhizosphere microbial communities.

## INTRODUCTION

Wetlands are globally impacted by agricultural industry through the leaching of nitrogen (N), mainly in the form of nitrate (NO_3_^−^), and by increased N deposition as a result of high emissions from fossil fuel burning and agriculture (1). Furthermore, due to reduced oxidation under stagnant, waterlogged conditions, these systems show increased availability of ammonium (NH_4_^+^) (2). The strongly increased anthropogenic N input influences ecosystem degradation by contributing to biodiversity loss and altering (mostly increasing) greenhouse gas fluxes such as nitrous oxide (N_2_O), methane (CH_4_), and carbon dioxide (CO_2_) (3–6).

The abundance, composition, and activity of microorganisms strongly influence the biogeochemical cycling of wetland nutrients, particularly those resulting in emissions of greenhouse gases (7, 8). Specifically, N_2_O emission may increase due to lowering of pH, which affects the activity of incomplete denitrifiers (4, 5, 9). CH_4_ emissions can increase due to competitive inhibition of the key enzyme of aerobic methanotrophs, methane monooxygenase (MMO), by elevated NH_4_^+^, osmotic stress of methanotrophs, or through the stimulation of methanogenic archaea (10–12). Finally, the rate of soil C loss can increase as a result of N addition through the stimulation of heterotrophic respiration (13). Although it is well established that microbial processes are important drivers of ecosystem functions, such as controls on greenhouse gas emissions and nutrient cycling, there is a lack of understanding of how these functions are linked, both to the environmental conditions and to the composition of the microbial community (8).

Wetland plant roots influence the soil region surrounding the root, known as the rhizosphere, by altering the availability of oxygen, organic matter, and organic plant exudates (14–16). The total area of soil influenced by roots can be considerable, meaning that this definition of the rhizosphere may extend to the vast majority of the upper soil layer (17). The rhizosphere is an active, complex ecosystem where viruses, bacteria, archaea, fungi, and protozoa interact with plant roots (18). These microorganisms significantly contribute to nutrient cycling and ecosystem structure by channeling energy into higher trophic levels (19, 20).

While the rhizosphere has been studied for decades, the effects of eutrophication on the plant-microbe interactions are of more recent interest. Specifically, it is important to understand how N availability influences plant physiology and ultimately C and N cycling in the rhizosphere. On the global scale, soil microbial communities differ, depending on the regional and local N regimes, although the diversity of these communities does not seem to vary much (21). Interestingly, variation in microbial community composition seems to be predictable based on local nutrient regimes (22, 23). Even though these studies demonstrate the link between nutrient loading and community structure, they do not demonstrate how changes in the microbial community are functionally relevant to the ecosystem.

To build dynamic models of plant-microbe interactions, it is necessary to gain a robust understanding of the connection between environmental conditions (i.e., N availability) and microbial community structure and function (i.e., the bulk biological processes resulting in greenhouse gas emissions). In this study, we aimed at assessing the impact of increased N input into wetland systems on the rhizosphere microbial community and its functions related to greenhouse gas production. To achieve this, we used *Juncus acutiflorus* (sharp-flowered rush), a very common graminoid plant in European wetlands that forms a dense vegetation and is known for radial oxygen loss (ROL) from roots (7). Furthermore, it has a high tolerance for increased N inputs (24). In the present report, a longitudinal study was used to determine that greenhouse gas emissions increase as a result of N addition in incubations with *J. acutiflorus*, but not in incubations with only bulk wetland soil, under controlled stable experimental conditions. Additionally, functional responses were linked to shifts in the dominant members of the microbial community. We hypothesize that certain key microbial groups contribute to greenhouse gas emissions, either directly or indirectly through the food web. Our study takes the first steps toward a predictive understanding of microbial dynamics within the rhizosphere, linking nutrient load, microbial community structure, and function.

## RESULTS

### Plant physiology.

*J. acutiflorus* and bulk soil were incubated over a course of 90 days under either a high-N treatment (800 kg N·ha^−1^·year^−1^) or a low-N treatment (40 kg N·ha^−1^·year^−1^). The soil collected from the Ravenvennen site and used in the incubations was a sandy soil with low organic matter content. Soil samples were taken at an initial time point (time zero [*T*_0_]), a midpoint (*T*_*m*_; *t* = 45 days), and final time point (*T*_*f*_; *t* = 90 days) (see Table S1 in the supplemental material). By *T*_*m*_, *J. acutiflorus* incubations had significant root development throughout the incubated soil such that all soil was dominated by root biomass. Thus, all soil sampled corresponded to the rhizosphere. To determine the N utilization of the plants and to identify growth responses to N inputs, the total dry weight biomass of roots, rhizomes, and shoots and total N and C contents of *J. acutiflorus* tissue were measured from plants at *T*_*f*_. Although there was no significant difference in total biomass and root/shoot ratio of *J. acutiflorus* between incubations, the average total N content of plant tissue (65 mg·g^−1^) was approximately twice as high in incubations with a high N input (*P* = 0.037) (see Table S2 in the supplemental material). Correspondingly, the total C/N ratio (averaged across the whole plant) was significantly higher in *J. acutiflorus* incubations with a low N input (*P* = 0.007) (Table S2). Interestingly, this change in C/N ratio was observed only for rhizome and shoot tissue, while the root C/N ratio did not significantly differ between incubations (Table S2).

10.1128/mSystems.00214-17.5 TABLE S1  Sample overview containing the time of sampling, N-load treatment, and whether the sample was bulk or rhizosphere soil. Additionally, the number of post-quality-filtered reads that were produced and the number of OTU found in each sample are shown. Greenhouse gas fluxes are reported in micromoles per square meter per day. Finally, Shannon diversity (*H*′) is reported. Download TABLE S1, XLSX file, 0.1 MB. Copyright © 2018 Hester et al. 2018 Hester et al. This content is distributed under the terms of the Creative Commons Attribution 4.0 International license.

10.1128/mSystems.00214-17.6 TABLE S2  Plant average dry weight (DW) and C/N ratio were determined in different sections of the plant, including the roots, shoots, and rhizomes. Biomass weight was determined as dry weight. The mean values from plants receiving high N and low N are reported (Mean High and Mean Low). The *P* value is reported as a result of a *t* test comparing mean values from high- and low-N treatments. Stdev, standard deviation. Download TABLE S2, XLSX file, 0.1 MB. Copyright © 2018 Hester et al. 2018 Hester et al. This content is distributed under the terms of the Creative Commons Attribution 4.0 International license.

### Greenhouse gas fluxes.

**FIG 1:**
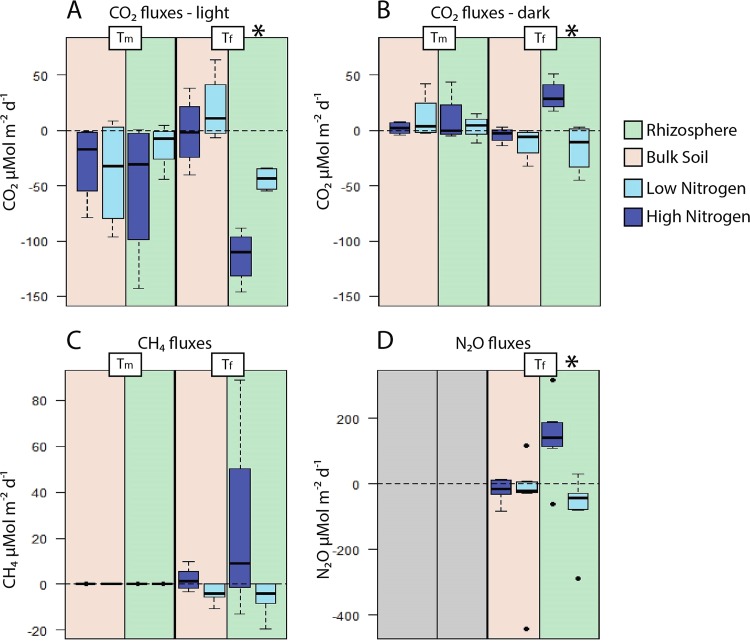
CO_2_, CH_4_, and N_2_O fluxes. Greenhouse gas fluxes were measured at a midpoint (*T*_*m*_) and the final time point (*T*_*f*_) during the 90-day incubation experiment. (A) CO_2_ light conditions, (B) CO_2_ dark conditions, (C) CH_4_, and (D) N_2_O. Asterisks denote significant differences (*P* < 0.05).

To link greenhouse gas fluxes with microbial community structure, gas flux measurements were performed at the same time points as soil sampling. Greenhouse gases were measured under both light and dark conditions, at *T*_*m*_ and *T*_*f*_ for CO_2_ and CH_4_ and at *T*_*f*_ for N_2_O (Fig. 1). Bulk soils generally did not have significant greenhouse gas fluxes (fluxes were not significantly different from 0) and will not further be discussed here. In the *J. acutiflorus* incubations, CO_2_ fluxes followed a day-night rhythm. Daytime CO_2_ fluxes were generally negative, indicating net CO_2_ fixation, with the largest rates significantly higher in high-N *J. acutiflorus* incubations at *T*_*f*_ (*t* = −5.28; *P* = 0.005) (Fig. 1A). Under dark conditions, CO_2_ fluxes were positive (net CO_2_ emission) only under the high-N treatment, while other treatments were not significantly different from 0 (*t* = 3.52; *P* = 0.01) (Fig. 1B). CH_4_ and N_2_O emissions did not vary between dark and light conditions, and therefore these conditions will not be compared. CH_4_ fluxes increased from *T*_*m*_ to *T*_*f*_, and emissions tended to be highest in the *J. acutiflorus* incubations with a high N input; however, there was large variability in this group (*t* = 2.165; *P* = 0.064) (Fig. 1C). N_2_O emissions were highest in the high-N treatment (*t* = 2.56; *P* = 0.04) (Fig. 1D), while a negative N_2_O flux was observed in *J. acutiflorus* incubations receiving a low N input (Fig. 1D), indicating that this system can function as an N_2_O sink under N-limited conditions.

### Denitrification potential.

To understand how increased N input influenced N cycling within bulk and *J. acutiflorus* rhizosphere soils, soil slurries were taken at *T*_*f*_ and their denitrification potential was measured. While we observed no significant difference in the N_2_ production between high- or low-N treatments (*t* = 0.32; *P* = 0.75), there was significantly higher N_2_O production from slurries originating from high-N-treatment soils (*t* = 2.41; *P* = 0.045) (see Fig. S2 in the supplemental material). This increased N_2_O production resulted in an approximately 10 times lower average N_2_/N_2_O ratio in high-N slurries (0.58 ± 0.61) compared to low-N-input slurries (5.36 ± 7.39), although not significantly different at *P* < 0.05 (*t* = −1.84; *P* = 0.11).

### Microbial community structure.

**FIG 2:**
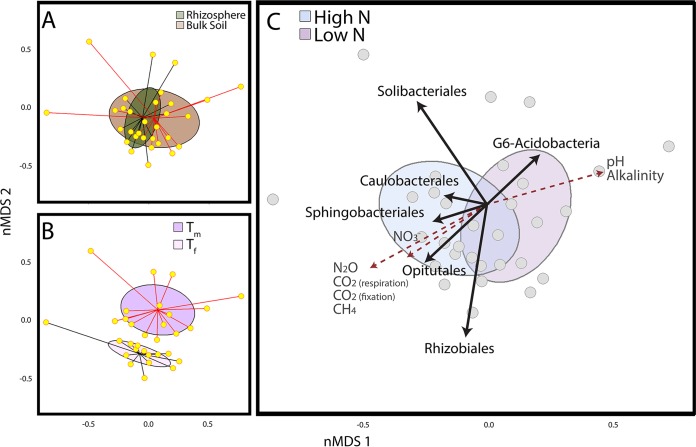
Microbial community structure and diversity. Nonmetric multidimensional scaling (nMDS) ordination plots of 16S rRNA samples show (A) rhizosphere or bulk soil, (B) the midpoint (*T*_*m*_) and the final time point (*T*_*f*_), and (C) high- and low-N treatment. The two-dimensional (2D) stress value was 0.19. Ellipses show the 95% confidence interval in the 2D space of samples in the respective treatment group. Red dashed lines indicate vectors for environmental parameters, while the black lines are for taxonomic groups.

The v3-v4 fragment of the 16S rRNA gene was amplified and sequenced resulting in, on average, over 1,100 post-quality control (post-QC) sequences per sample. Each sample contained on average 264 ± 136 (mean ± standard deviation [SD]) operational taxonomic units (OTU). Rarefaction curves (see Fig. S3 in the supplemental material) suggest that sequencing depth was insufficient to capture the complete diversity of the communities. However, sampling depths of individual communities were not statistically linked to particular experimental groups, suggesting that there was a minimal effect of sampling effort on the group comparisons (N, *P* = 0.46; time, *P* = 0.19; rhizosphere, *P* = 0.69; *t* test) and that the observed community changes were caused by the different incubation conditions. In addition, over the course of the experiment the overall composition of communities changed (*P* < 0.001; permutational multivariate analysis of variance [PERMANOVA]) (Fig. 2B). There were also significant differences in the community composition of high- and low-N-treatment incubations (*P* = 0.003; PERMANOVA) (Fig. 2C) as well as rhizosphere and bulk soil incubations (*P* = 0.02) (Fig. 2A).

On average, microbial diversity increased between *T*_*m*_ and *T*_*f*_ (*t* = 2.516; *P* = 0.0176; Shannon diversity index [*H*′]) (Table S1). Within each time point, diversity did not differ significantly between *J. acutiflorus* and bulk soil incubations, nor did N input have an impact (Table S1).

### Linking microbial community members to function.

In order to understand how the microbial community members were linked to environmental conditions and greenhouse gas emissions, a random forest classifier was used to identify microbial taxa whose abundance was affected by N input, time of sampling, or presence of *J. acutiflorus*. Additionally, random forest was also used for regression to determine connections between abundance of these groups and environmental conditions or greenhouse gas fluxes, and these associations were further analyzed by fitting linear models.

The top three microbial groups that significantly responded to N input were the *Opitutales* (*Verrucomicrobia*) and *Sphingobacteriales* (*Bacteroidetes*), which were more abundant in the high-N-treatment group, and group 6 (G6) *Acidobacteria*, which were more abundant in the low-N-treatment group (Fig. 2C; Table 1). More specifically, the relative abundances of these three orders could be linked to N_2_O emissions (Table 1). *Opitutales* and *Sphingobacteriales* were positively associated with N_2_O fluxes, while a negative association was observed for the G6 *Acidobacteria*. In addition, *Sphingobacteriales* were correlated to CO_2_ fixation (Table 1).

**TABLE 1:** Correlations of microbial community members to environmental conditions and greenhouse gas fluxes^a^

Microbial community and parameter	*t*	*P* value	Mean relative abundance		Correlate	Adjusted *R*^2^ value	Coefficient	*P* value
High versus low N			High N	Low N				
*Opitutales*	4.17	<0.001	0.040	0.010	N_2_O	0.11	3.50E−4	0.012
G6 *Acidobacteria*	−4.22	<0.001	0.007	0.020	N_2_O	0.19	−3.18E−5	0.058
*Sphingobacteriales*	2.88	0.008	0.010	0.005	N_2_O	0.32	3.10E−5	0.016
					CO_2_ (fixation)	0.29	7.07E−5	0.011
Rhizosphere vs bulk soil			Rhizosphere	Bulk				
*Caulobacterales*	−3.46	0.002	0.052	0.032	NO_3_^−^	0.21	−8.50E−5	0.003
*T*_*m*_ vs *T*_*f*_			*T_m_*	*T_f_*				
*Rhizobiales*	6.66	<0.001	0.099	0.184	CO_2_ (respiration)	0.27	−6.40E−4	0.001
*Solibacterales*	−4.76	<0.001	0.179	0.116	Alkalinity	0.26	−2.00E−2	0.002

a The mean relative abundances of the top bacterial families distinguishing high versus low N, rhizosphere versus bulk soil or *T*_*m*_ versus *T*_*f*_ sampling time points are indicated, as are the *t* test results and statistics. Additionally, the top environmental or functional traits correlated with these groups are reported along with linear model statistics.

The top bacterial order distinguishing microbial communities from rhizosphere and bulk soil were the alphaproteobacterial order *Caulobacterales*, which were more abundant in the rhizosphere than in bulk soil and had a negative association with elevated NO_3_^−^ concentrations (Fig. 2A and C; Table 1). The *Rhizobiales* and *Solibacterales* orders of the *Alphaproteobacteria* class and *Acidobacteria* phylum, respectively, were most distinctive for the microbial communities sampled at *T*_*m*_ versus *T*_*f*_ (Fig. 2B; Table 1). *Rhizobiales* abundance was negatively associated with CO_2_ fluxes under dark conditions, while the *Solibacterales* were correlated to pore water alkalinity, which is a proxy for anaerobic decomposition (25) (Fig. 2; Table 1).

### Soil metagenomics.

In addition to sequencing the 16S rRNA genes, which do not allow inference of an organism’s functional traits on their own, total DNA was sequenced from 5 soils derived from *T*_0_ and rhizosphere and bulk soil samples at *T*_*m*_ and *T*_*f*_ from the high-N treatment. The goal of the metagenomic sampling was to survey the genetic potential of organisms that were most strongly influenced by N loading (i.e., those identified in the 16S rRNA analysis). In particular, we wanted to find support for the roles the above taxa have in the rhizosphere of *J. acutiflorus*. These libraries resulted in on average 1 million post-QC reads per library (see Table S3 in the supplemental material). Coassembly of the metagenomic reads from all sequencing libraries yielded over 130,000 contigs with a mean contig length of 1,053 bp and a maximum length of over 46 kbp. The contigs were binned to obtain metagenome-assembled genomes (MAGs), with subsequent taxonomic assignment and genome completeness estimation. Bins with taxonomic affiliations matching with the taxa identified above as being associated with different N treatments were used for further analysis. Of the three selected bins, the *Acidobacteria* bin consisted of 261 contigs (assembled with 5,198 mapped reads), the *Opitutales* bin 374 contigs (3,979 reads), and the *Sphingobacteriales* bin 164 contigs (10,111 reads), with genome completeness estimates of 2.04, 12.25, and 2.35%, respectively. The *Acidobacteria*, *Opitutales*, and *Sphingobacteriales* contigs had *N*_50_ scores of 2,221, 1,897, and 2,856 bp, respectively.

10.1128/mSystems.00214-17.7 TABLE S3  Metagenome library overview, including the time of sampling (Time), number of post-QC reads (Reads), average length (Avg_len), and the standard deviation in read length (Sd_len). Download TABLE S3, XLSX file, 0.1 MB. Copyright © 2018 Hester et al. 2018 Hester et al. This content is distributed under the terms of the Creative Commons Attribution 4.0 International license.

Although estimated to be highly incomplete, all bins were annotated to identify the functional potential of these species. The *Acidobacteria* bin contained carbon metabolism-associated genes involved in polysaccharide degradation and in the anaerobic degradation of aromatic compounds. Other than a nitrate-nitrite transporter, no nitrogen cycling genes were detected. The *Opitutales* bin contained a diverse set of genes related to oligosaccharide degradation and fermentation (acetoin and butyryl-coenzyme A [CoA] dehydrogenase), an amylomaltase for polysaccharide degradation, and genes for organic acid utilization. In addition, multiple fatty acid-, lipid-, and isoprenoid biosynthesis-related genes were detected. Among the nitrogen cycling genes detected were genes associated with nitrogen fixation (nitrogenase), denitrification (nitrous oxide reductase), and hydroxylamine reduction. The *Sphingobacteriales* bin contained carbon metabolism genes associated with di- and oligosaccharide degradation and fermentation (sugar/maltose fermentation stimulation protein homolog), a xylanase, and genes involved in the utilization of xylose as well as other plant-associated one-carbon metabolism-related genes.

As is reflected by the low completeness estimations and highly fragmented nature of our MAGs, retrieval of high-quality genomes from soil metagenomic data sets is highly challenging. To circumvent these challenges, we additionally applied a gene-centric approach to survey genetic potential for N and C cycling in N amended samples. Custom databases of genes involved in N and C cycling processes (26) were used to identify metagenomic reads of major N cycling genes (*amoA* and *hao*, involved in NH_4_^+^ oxidation; *narG*, *nirK*, *nirS*, *norB*, and *nosZ*, involved in denitrification; *nrfA*, involved in dissimilatory nitrite reduction to ammonia; and* nifH*, involved in N fixation) and CH_4_ cycling genes (*pmoA* and *mmoX*, involved in CH_4_ oxidation; and *phnGHI* and *mcrA*, involved in methanogenesis) and their abundance in the high-N incubations (abbreviations found in Table S4 in the supplemental material). There were no *nirS* genes detected in the data set, and only two reads annotated as *mcrA* were detected in the metagenomes. All other N and CH_4_ cycling genes were present (see Table S5 in the supplemental material).

10.1128/mSystems.00214-17.8 TABLE S4  N and C cycling gene abbreviations. Download TABLE S4, XLSX file, 0.1 MB. Copyright © 2018 Hester et al. 2018 Hester et al. This content is distributed under the terms of the Creative Commons Attribution 4.0 International license.

10.1128/mSystems.00214-17.9 TABLE S5  The number of reads for each N and C cycling marker gene and their respective relative abundances (expressed in parentheses) in soil/rhizosphere metagenomes. Download TABLE S5, XLSX file, 0.1 MB. Copyright © 2018 Hester et al. 2018 Hester et al. This content is distributed under the terms of the Creative Commons Attribution 4.0 International license.

## DISCUSSION

Greenhouse gas emissions remain a global challenge. A thorough understanding of the factors that alter microbial community structure and function, such as increased N input, is important in developing management strategies for greenhouse gas emissions. This is particularly important in ecosystems as extensive as wetlands. With an estimated area of up to 12.8 million km^2^ worldwide, wetlands considerably contribute to the total terrestrial carbon storage (27, 28). Here we studied the impact of increased N input on the microbial community and greenhouse gas fluxes from the rhizosphere of *J. acutiflorus*, a very common plant in European wetland ecosystems and a model for other *Juncus* species globally. We found characteristic shifts in the microbial community structure and a stimulation of greenhouse gas fluxes in *J. acutiflorus* incubations in response to N input.

### Plant physiological shifts as a response to high-N inputs.

**FIG 3:**
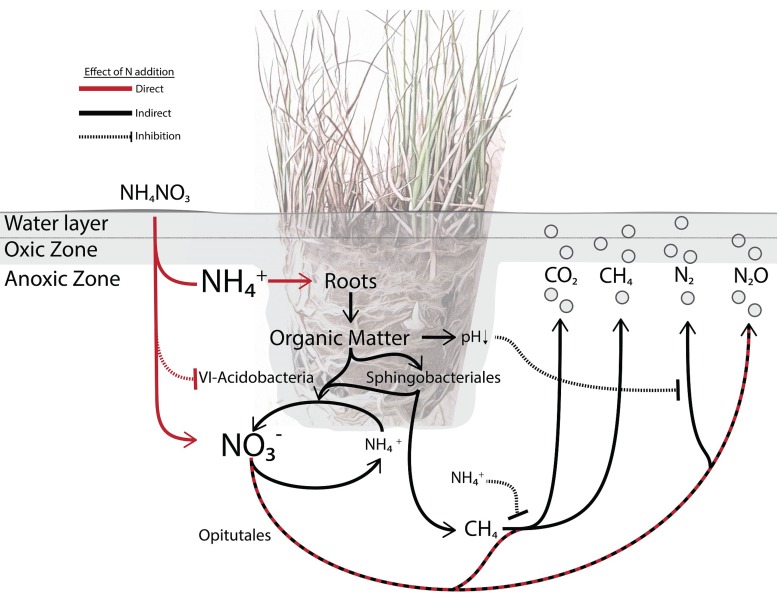
A *Juncus acutiflorus* rhizosphere microbial food web model. In the model, microbial processes are directly (red lines) or indirectly (black lines) influenced by N deposition. *J. acutiflorus* preferentially takes up NH_4_^+^, which stimulates plant productivity and rhizodeposition of organic matter and oxygen (24). Released oxygen and labile organic matter contribute to soil acidification, in addition to stimulating complex polymer degradation (*Sphingobacteriales*) and heterotrophic denitrifiers (*Opitutales*). The production of N_2_ can be affected by a drop in pH, which influences the activity of complete denitrifiers. The group 6 *Acidobacteria* are outcompeted at higher N availability. Recalcitrant organic matter degraded by *Sphingobacteriales* can enter the microbial food web and be fermented by fermenters, which in turn provide substrates for methanogens (*mcr*). The activity of phosphonate lyases (*phn*) might also stimulate the production of methane, while anaerobic methane oxidation also contributes to methane consumption. Additionally, methane consumption by aerobic methanotrophs through methane monooxgenases (*pmo*) could be inhibited by excess NH_4_^+^ (12).

The plant plays a prominent role in the maintenance of the rhizosphere microbial community (29). Roots release oxygen through radial oxygen loss, providing an oxic niche in otherwise anoxic wetland soils (30). Plants also release labile organic matter in the form of organic acids, neutral sugars, and amino acids (31, 32). The composition of this organic matter structures the microbial community within the rhizosphere by providing different substrates for heterotrophic microorganisms (33). The exuded organic acids acidify the surrounding soil, preventing many microbial species from thriving within the rhizosphere, but also modifying nutrient availability (34, 35). The quantity of organic matter released is closely associated with photosynthetic activity (36). As plants are often N limited in natural systems, relieving this limitation promotes plant growth (37). In this study, we observed that when incubated under high-N input, *J. acutiflorus* showed increased C fixation rates (Fig. 1A) and plant tissue becomes saturated with N (Table S2). This also suggests that *J. acutiflorus* without N limitation excretes larger amounts of labile carbon into the surrounding soil, which is also evident from the observed decreases in pore water pH in the high-N incubations (see Fig. S4 in the supplemental material). Additionally, due to root-derived oxygen, increased nitrification rates could contribute to this observed drop in pH (7). Together, higher N input could result in higher photosynthetic rates in *J. acutiflorus* specimens, likely depositing larger amounts of organic matter into surrounding soil, stimulating the heterotrophic microbial community in return (Fig. 2 and 3).

### Greenhouse gas fluxes as a result of N input.

N availability has previously been shown to alter greenhouse gas emission dynamics (8). Here we observed that greenhouse gas fluxes, both positive and negative, in *J. acutiflorus* incubations were stimulated by increased N input (Fig. 1). CO_2_ fixation rates were highest in *J. acutiflorous* incubations with high-N input under the light conditions, likely due to increased photosynthetic activity of the plant and photosynthetic microorganisms. In the dark, the same *J. acutiflorus* incubations showed elevated CO_2_ emissions, likely due to increased plant and microbial respiration (Fig. 1A and B). The highest CH_4_ emissions were observed in *J. acutiflorus* incubations with high-N input, although with large variability (Fig. 1C). Still, the elevated emission rates suggest that the *J. acutiflorus* rhizosphere could become a net source of CH_4_ under high-N input. The total amount of CH_4_ released reflects the sum of CH_4_ production (methanogenesis) and consumption (methanotrophy). In the present study, both *mcrA* and *phnGHI* genes, which are involved in the production of methane, as well as *pmoA* and *mmoX*, involved in methane oxidation, were detected (Table S5). Methanogenesis has been linked to plant productivity, thought to be due to increased availability of labile organic carbon from photosynthate exudates (38, 39). Furthermore, methanogens can be stimulated through an indirect priming mechanism. Labile organic matter from plant exudation can stimulate microbial activity responsible for degrading recalcitrant organic matter, which in turn makes this carbon source available to methanogens (40–43). Alternatively, net CH_4_ emissions can be increased by inhibiting CH_4_ consumption—for instance, through the competitive inhibition of the key enzyme methane monooxygenase by NH_4_^+^ (44, 45).

The reduction of NO_x_ to N_2_ is often incomplete, resulting in the production of the greenhouse gas N_2_O. Incomplete denitrification occurs when microbial species do not utilize N_2_O as an electron acceptor either due to physiological constraints or induced by certain environmental conditions (46, 47). It has been observed that N fertilization has the largest impact on N_2_O emission, with NO_3_^−^ availability being the main driver of emission rates (4). As denitrification is largely a microbial process, the composition of the microbial community plays an important role in the total amount of gaseous N forms emitted from soils. Representatives from a diverse set of phyla are known to denitrify (8, 46), and denitrification rates are therefore considered to be robust to changes in the microbial community composition (48). Here we observed elevated N_2_O emissions in *J. acutiflorus* incubations under high-N input, whereas they acted as an N_2_O sink in the low-N incubations (Fig. 1D). Interestingly, N_2_O emissions by bulk soil were not significantly influenced by the tested N regimes, despite the microbial community containing the full suite of denitrification-associated genes, indicating that *J. acutiflorus* plays a substantial role in stimulating N-reducing microbial species, probably by supplying labile carbon (Fig. 1D; Table S5). Elevated N loading additionally caused an almost 10-fold increase in the production of N_2_O relative to N_2_, suggesting that high-N input can shift the microbial rhizosphere community toward partial denitrifiers, which has important implications given the strong greenhouse potential of N_2_O.

### Shifts in microbial community structure as a response to N input.

Association of microbial metabolisms (i.e., those resulting in greenhouse gas emission) with the structure of microbial communities and abiotic factors defined by the environment is essential to predict how the structure and function of these microbial ecosystems may adapt to future conditions. Bulk and rhizosphere soils contain diverse microbial communities with equally diverse metabolisms (49, 50). It remains a challenge to understand the role that key groups play in these systems and how they affect their environment.

Using 16S rRNA gene amplicon sequencing, we were able to identify three bacterial orders that were associated most strongly with N input and greenhouse gas emissions (Fig. 2C; Table 1). We further investigate the potential functional roles of these species in the *J. acutiflorus* rhizosphere by utilizing metagenomic data. Due to the immense diversity of the soil ecosystem, it is challenging to recover high-quality genomes from these systems. As a result, we were only able to obtain partial genomes of the organisms identified in the amplicon data set. We therefore built a conceptual model of the *J. acutiflorus* rhizosphere by first utilizing our metagenomic data, followed by conservatively drawing support from available literature on these organisms.

The verrucomicrobial *Opitutales* were associated with high-N input and elevated N_2_O emissions. Members of this order are diversely associated with different rhizospheres, ranging from sugarcane to wetland plants (51, 52). They have been physiologically described as anaerobic polysaccharide-utilizing bacteria that are capable of reducing NO_3_^−^ to NO_2_^−^ (53). In the present study, the *Opitutales* bin contained a diverse array of carbohydrate-degrading and fermentation-associated genes, including genes encoding an amylomaltase, which catalyzes the transglycosylation of maltodextrins, and butyryl-CoA and acetoin dehydrogenases. Apart from the O_2_ derived from the plant roots, which is quickly consumed by aerobic heterotrophs, wetland soils are waterlogged systems resulting in an anoxic environment. *Opitutales* may take advantage of the abundant organic carbon in the rhizosphere and anoxic niches, possibly fermenting some fraction of this carbon pool. While many of the carbon-related genes detected in the *Opitutales* bin and the nitrous oxide reductase are consistent with the literature, we did not detect nitrate or nitrite reductase as expected. A lack of genome completeness prevents conclusive statements about the role this particular *Opitutales* group plays in the *J. acutiflorus* rhizosphere; however, it is probable that expected denitrification genes were not retrieved. Based on a combination of literature describing the physiology of *Opitutales* from other rhizosphere environments with genomic evidence from the present study, it is probable that members of this group are taking advantage of plant-derived organic matter and have a denitrifying potential (Fig. 3).

The *Sphingobacteriales* from the phylum *Bacteroidetes* were also overrepresented in the high-N-input incubations (Fig. 2; Table 1). Described *Sphingobacteriales* are understood as copiotrophic bacteria, referring to their ability to metabolize a wide array of carbon sources and being present at great abundances in soils with high carbon availability (54, 55). Consistent with this, the *Sphingobacteriales* bin from the present study contained a wide array of genes encoding enzymes involved in carbohydrate utilization, particularly sugars that would originate from the plant, such as xylose. In the present study, the majority of organic matter would originate from the plant as the sandy soil used had low organic matter content. Rhizodeposition in this case would be very important for microbial groups such as *Sphingobacteriales*, not only as a carbon source but also as an O_2_ source, since *Sphingobacteriales* have been reported to be particularly sensitive to O_2_ availability. When tested for cellulolytic activity in oxic or anoxic environments, they were exclusively active in oxic treatments, suggesting that this group may require oxygenated environments for at least some forms of carbon degradation (56). Interestingly, the *Sphingobacteriales* bin contains some fermentation-associated genes (i.e., sugar/maltose fermentation stimulation protein homolog), indicating possible flexibility in oxygen requirements. Considering findings from this study and the literature, we hypothesize that *Sphingobacteriales* within the *J. acutiflorus* rhizosphere could be benefiting from the elevated carbon input from the roots under higher N input, cycling this carbon and possibly making it available to other community members (Fig. 3).

G6 *Acidobacteria* were overrepresented in the low-N-input incubations, and there was no significant difference in their abundance between bulk and rhizosphere soils. Unlike *Opitutales* and *Sphingobacteriales*, they were negatively correlated with N_2_O emissions (Fig. 2; Table 1). While the G6 *Acidobacteria* group is not well studied, one genome (GenBank accession no. CP015136.1) was recently published (57) and was shown to contain nitric and nitrous oxide reductases. In the *Acidobacteria* bin, a nitrate-nitrite transport gene was detected; however, no denitrification-associated genes were present. Genomic and physiological studies of a closely related group (group 1 *Acidobacteria*) showed that they were anaerobic organoheterotrophs capable of utilizing NO_3_^−^ for respiration (58), and other *Acidobacteria* have also been described as important soil carbon and N cyclers. However, many N-cycling reactions are restricted to particular clades, indicating that these functions are heterogeneously represented across the *Acidobacteria* phylum (59, 60). In addition to N cycling, *Acidobacteria* are known for their utilization of C derived from autotrophic microorganisms in anoxic environments (61). They have been reported to utilize various plant- and microbe-derived polysaccharides, like xylan, cellobiose, and gellan (60, 62), and thrive in various soils and rhizospheres, including anoxic soils with low pH (54, 63). We detected multiple genes associated with carbohydrate metabolism in the *Acidobacteria* bin, namely, those for maltose and maltodextrin utilization. The cultured representatives of *Acidobacteria* have low growth rates and appear to be adapted to oligotrophic environments (54, 64). Thus, G6 *Acidobacteria* may not be competitive under high N availability with fast-growing (partial) denitrifiers. Together, in the low-N-input rhizosphere, the G6 *Acidobacteria* may be involved in a slower turnover of organic carbon, either from other bacteria in the community or from plant biomass. Increased N availability might reduce this group’s abundance by altering competitive advantages (Fig. 3).

### A model microbial food web within bulk soil and the *J. acutiflorus* rhizosphere.

Increased N input poses a distinct threat to wetland ecosystems, contributing to the degradation of biodiversity and altering greenhouse gas emissions (3, 8). Plants such as *J. acutiflorus* influence the abundance and composition of microorganisms living in the rhizosphere by exuding organic matter and releasing oxygen from their roots (29). In the present study, N addition resulted in increased productivity of *J. acutiflorus*, stimulating the effect of the plant on the microbial community but also directly affecting microbial metabolism. Based on our observations and published knowledge, we built a conceptual model of the *J. acutiflorus* microbial food web, indicating how N input impacts the soil microbial community (Fig. 3).

N fertilization can influence the soil microbial community by providing excess NH_4_^+^ and NO_3_^−^. Previous studies have shown that *J. acutiflorus* prefers NH_4_^+^ over NO_3_^−^ as an N source, leading to a surplus of NO_3_^−^ in the rhizosphere (24) (Fig. S4). This alters N cycling dynamics in the rhizosphere, favoring microbial species and metabolisms reducing NO_3_^−^ to N_2_O rather than to N_2_ (65, 80) (Fig. 1; Fig. S2). The combined effect of enhanced plant-derived carbon input and higher N availability stimulates heterotrophic activity, resulting in increased N_2_O and CO_2_ emissions (Fig. 1 and 3). While excess NO_3_^−^ spurs anaerobic respiration, increased NH_4_^+^ concentrations can lead to an inhibition of methane oxidation, possibly contributing to the heterogeneity observed in CH_4_ emissions (Fig. 1C). High N availability can also have an indirect effect by influencing plant physiology. The observed increased rates of carbon fixation by *J. acutiflorus* under high-N input may result in augmented release of organic matter (including organic acids) and oxygen from the roots. This acidifies the rhizosphere soil, which can alter the activity of *nosZ*-containing microbes (65). Additionally, elevated oxygen availability stimulates heterotrophic activity in an otherwise anoxic environment, leading to higher CO_2_ emissions. Thus, altered N input in the *J. acutiflorus* rhizosphere leads to increased greenhouse gas fluxes directly by altering the abundance of N-cycling species and indirectly through the stimulation of plant primary productivity (Fig. 3).

### Conclusions.

With continued anthropogenic inputs of nitrogen into wetlands, it is critical to understand how this activity may affect globally relevant carbon and nitrogen cycling within wetlands. The results here support that under high N input, greenhouse gas emissions from the *J. acutiflorus* rhizosphere increase, shifting the system from a greenhouse gas sink to a source. Three bacterial orders, the *Opitutales*, G6 *Acidobacteria*, and *Sphingobacteriales*, respond to increased N availability, and genomic evidence supports their involvement in processes leading to changes in greenhouse gas fluxes. Our view is that understanding interactions within the rhizosphere that result in increased greenhouse gas emissions is essential for creating management solutions aimed to address emission goals, efficient agricultural practices, and conservation efforts. To move forward in our understanding of the complex dynamics within ecosystems such as the rhizosphere, future effort needs to be made in building extensive data sets that can be used to build predictive models of how these microbial ecosystems might respond under altered environmental conditions. We propose that conceptual models, such as our *J. acutiflorus* rhizosphere plant-microbial food web model, should be used to set the framework for building such data sets.

## MATERIALS AND METHODS

### Sample collection and experimental setup.

Plants and sandy soil were sampled from the Ravenvennen (51.4399°N, 6.1961°E) in Limburg, The Netherlands (August 2015), and returned to the Radboud University greenhouse facilities for conditioning. The Ravenvennen is a protected marshy area consisting of sandy soil rich in vegetation with a high prevalence of *Juncus* spp. Plants were removed from soil, and rhizomes were cut into eight 2-cm fragments and reconditioned on hydroculture in a nutrient-rich medium as described by Hoagland and Arnon (66). After sufficient root development (to approximately 25 cm after 2 weeks), eight plants and eight bulk soil incubations were randomly assigned to high- or low-nitrogen experimental groups, totaling 16 incubations (see Table S1 and Fig. S1 in the supplemental material). Soil collected from the field was homogenized and sieved to remove any contaminating roots and potted. The reconditioned plants were transferred to pots with diameters of 19 cm at the base and 26 cm at the top and a height of 19 cm containing the prepared soil, moved to an indoor water bath set to 15°C with a cryostat (Neslab Thermoflex 1400; Thermo Electron Corp., Breda, The Netherlands), and cultivated with a day/night cycle of 16 h of light and 8 h of dark (Master Son-T PiaPlus; Philips, Eindhoven, The Netherlands). Pots were kept waterlogged with a 2-cm water layer on top. A drip-percolation-based system ensured a constant supply of nutrients. The low-N-input nutrient solution contained 12.5 µM NH_4_NO_3_, corresponding to an N loading rate of 40 kg N·ha^−1^·year^−1^. The high-N-input solution contained 250 µM NH_4_NO_3_, corresponding to 800 kg N·ha^−1^·year^−1^. These rates fall within N loading of wetlands in agricultural catchments and thus represent contrasting extremes (67).

10.1128/mSystems.00214-17.1 FIG S1  Experimental design schema depicting sample replicates per treatment in either rhizosphere or bulk soil. Additionally, the sampling points and types are denoted by colored boxes. GHG, greenhouse gas. Download FIG S1, EPS file, 1.3 MB. Copyright © 2018 Hester et al. 2018 Hester et al. This content is distributed under the terms of the Creative Commons Attribution 4.0 International license.

10.1128/mSystems.00214-17.2 FIG S2  Denitrification potential from soil slurries. N_2_ and N_2_O production rates were estimated to determine potential denitrification of the soil and rhizosphere microbial communities. Download FIG S2, EPS file, 2.7 MB. Copyright © 2018 Hester et al. 2018 Hester et al. This content is distributed under the terms of the Creative Commons Attribution 4.0 International license.

10.1128/mSystems.00214-17.3 FIG S3  Rarefaction curves with number of species observed as a function of sequencing effort (sample depth). Download FIG S3, EPS file, 1.8 MB. Copyright © 2018 Hester et al. 2018 Hester et al. This content is distributed under the terms of the Creative Commons Attribution 4.0 International license.

10.1128/mSystems.00214-17.4 FIG S4  Pore water inorganic nutrients, pH, and alkalinity. Shown are the concentrations of inorganic nutrients, pH, and alkalinity in pore water sampled throughout the incubation. Download FIG S4, EPS file, 2.8 MB. Copyright © 2018 Hester et al. 2018 Hester et al. This content is distributed under the terms of the Creative Commons Attribution 4.0 International license.

### Incubation measurements.

Five representative *J. acutiflorus* specimens were harvested for initial measurements of plant dry weight and C/N ratios. At the final time point (*T*_*f*_, 90 days), all plants were harvested to measure dry weight and C/N ratios of roots, shoots, and rhizomes. Pore water was extracted using 0.15-µm porous soil moisture samplers (SMS rhizons, Rhizosphere Research Products, Wageningen, The Netherlands) and measured over the course of the experiment to determine inorganic nutrients as well as metals using an AutoAnalyzer (AutoAnalyzer 3; Bran+Luebbe, Germany) and ICP-OES (iCAP6000; Thermo Scientific, Waltham, MA). To reduce the impact of soil heterogeneity, samples were extracted in duplicate and mean values were calculated.

### Greenhouse gas measurements.

To determine greenhouse gas fluxes, a cylindrical transparent collection chamber (7.5 by 30 cm) was used to measure accumulation or depletion of CO_2_, CH_4_, and N_2_O in the headspace. CO_2_ and CH_4_ fluxes were measured at *T*_*m*_ (45 days), and *T*_*f*_ and N_2_O fluxes were measured at *T*_*f*_. Fluxes were measured using a Picarro G2308 NIRS-CRD greenhouse gas analyzer (Picarro, Inc., Santa Clara, CA). Fluxes were determined by fitting a smoothed spline to the time series using the R function *sm.spline* from the *pspline* package, and the average rate of change was calculated (68).

### Denitrification potential.

To measure denitrification potential, two soil slurries were made from each experimental pot by mixing 50 g soil with 100 ml Milli-Q water, divided into control and experimental bottles, and made anoxic by flushing with argon gas. Bottles were preincubated overnight at 15°C to allow for residual unlabeled NO_3_^−^ to be consumed. A ^15^N-labeled NaNO_3_ solution was added to the experimental bottles to a final concentration of 500 µM, and a KCl solution was added to the control bottles to a final concentration of 500 µM. Production of N_2_O and N_2_ was measured by taking samples 2, 7, and 22 h after adding substrate on a gas chromatography-mass spectrometry (GC-MS) device (5975C; Agilent Technologies, Santa Clara, CA).

### DNA extraction and 16S rRNA amplicon and metagenomic sequencing.

Soil was collected from each of the 16 incubations at three time points: one initial soil sample from the site and *T*_*m*_ and *T*_*f*_ samples. A single core per pot was taken using a 1- by 7-cm corer. DNA was extracted using the PowerSoil DNA isolation kit (MoBio, Carlsbad, CA). From all 16 incubations, 16S rRNA genes (variable regions 3 to 4) were amplified in triplicate reactions using IonTorrent sequencing adapter barcoded primers 341F (CCATCTCATCCCTGCGTGTCTCCGACTCAGxxxxxxxxxxGATCCTACGGGNGGCWGCAG) and 785R (CCACTACGCCTCCGCTTTCCTCTCTATGGGCAGTCGGTGATGACTACHVGGGTATCTAATCC) and pooled. The pooled amplicons were cleaned with Ampure beads (Beckman Coulter, Inc., Fullerton, CA) and subsequently prepared for sequencing on the IonTorrent PGM using the manufacturer’s instructions (Life Technologies, Inc., Carlsbad, CA).

From the same DNA samples, total DNA from 5 representative incubations (4 *T*_*m*_, 4 *T*_*f*_, 16 *T*_*m*_, and 16 *T*_*f*_ samples and the initial soil sample) was sheared into approximately 400-bp fragments via sonication. The resulting fragments were prepared for sequencing following the manufacturer’s instructions with the Ion Plus Fragment Library kit (Life Technologies, Inc., Carlsbad, CA).

### Data analysis.

16S rRNA gene amplicons were filtered for quality (*Q* > 25) and size (>200 bp) using QIIME v1.9 (69). Quality-controlled reads were then clustered into OTU at a 97% identify level and phylogenetically classified by utilizing the NINJA-OPS v1.3 pipeline (70). The reference database used for taxonomic assignment was the SILVA database version 123 (71). The resulting OTU table was used for downstream analysis in R (72). Count data were normalized to relative abundances to account for differing sequence depth between samples, and a square root transformation was applied. The *vegan* R package was used to calculate Shannon diversity with the *diversity* function, Bray-Curtis dissimilarity matrices with the *vegdist* function, permutational multivariate analysis with the *adonis2* function, and nonmetric multidimensional scaling (nMDS) with the *metaMDS* function (73). The Bray-Curtis dissimilarity was used in calculating the nMDS. The *RandomForest* R package was used for classification and regression (74). Linear models were fit with the *glm* function in the *stats* package.

Metagenomic reads were quality filtered (*Q* > 25), and small fragments (<100 bp) were removed using PrinSeq (75). Quality-filtered reads were assembled using metaSPAdes (version 3.7 [76]). The Resulting contigs (>1 kbp) were binned using BinSanity, and taxonomic assignments and bin quality were checked with CheckM (77, 78). Annotations of bins were performed using the SEED database (79).

### Accession number(s).

Raw reads were submitted to NCBI and archived under SRA accession no. SRP099838.
